# Combinatorial approaches for inverse metabolic engineering applications

**DOI:** 10.5936/csbj.201210021

**Published:** 2013-03-11

**Authors:** Georgios Skretas, Fragiskos N. Kolisis

**Affiliations:** a Institute of Biology, Medicinal Chemistry and Biotechnology, National Hellenic Research Foundation, Athens, Greece; b Biotechnology Laboratory, School of Chemical Engineering, National Technical University of Athens - Zografou Campus, Athens, Greece

**Keywords:** inverse metabolic engineering, genetic engineering, microbes, genetic screening, mutagenesis

## Abstract

Traditional metabolic engineering analyzes biosynthetic and physiological pathways, identifies bottlenecks, and makes targeted genetic modifications with the ultimate goal of increasing the production of high-value products in living cells. Such efforts have led to the development of a variety of organisms with industrially relevant properties. However, there are a number of cellular phenotypes important for research and the industry for which the rational selection of cellular targets for modification is not easy or possible. In these cases, strain engineering can be alternatively carried out using “inverse metabolic engineering”, an approach that first generates genetic diversity by subjecting a population of cells to a particular mutagenic process, and then utilizes genetic screens or selections to identify the clones exhibiting the desired phenotype. Given the availability of an appropriate screen for a particular property, the success of inverse metabolic engineering efforts usually depends on the level and quality of genetic diversity which can be generated. Here, we review classic and recently developed combinatorial approaches for creating such genetic diversity and discuss the use of these methodologies in inverse metabolic engineering applications.

## 1. Introduction

Metabolic engineering has been a well established scientific discipline for over two decades now [1]. During this period, advances in the understanding of the functional role of thousands of genes in various organisms, and the development of theoretical and experimental tools for determining the flow of metabolites through different biochemical pathways [2, 3], have provided hints for numerous potential cellular targets whose modification can lead to optimal metabolism and improved properties.

More recently, advances in sequencing technologies have resulted in an explosion in the number of sequenced microbial genomes, revealing a plethora of novel enzymes, biochemical reactions and pathways, while the development of efficient methodologies for performing directed protein evolution has enabled the engineering of enzymes with tailored activities [4, 5]. Furthermore, the emergence of more global approaches for analysing cell function, such as systems biology, have contributed to our understanding of how biochemical pathways operate, not in the form of sequential “isolated” reactions, but instead as complex, interdependent and dynamic networks of such reactions [6]. Finally, synthetic biology has extended the capabilities of classical metabolic engineering as it has provided the rationale and tools to combine biological components and generate designed gene networks, artificial metabolic pathways, and organisms with man-made genomes [7, 8].

These technologies, in combination with the significantly advanced analytical tools available today for analyzing levels of DNA, RNA, proteins and metabolites in the cell, have enabled the development of engineered prokaryotic and eukaryotic organisms with the ability to produce a wide array of chemicals useful for research and the industry: amino acids [9], antibiotics [10], alkaloids [11], isoprenoids [12], vitamines, fragrances, peptides and polyketides [13], organic solvents and biofuels [14, 15], nutraceuticals, polymers [16] and others.

A prerequisite for the success of classical (forward) metabolic engineering is a detailed knowledge of the biochemical pathways involved in the biosynthesis of a particular metabolite or in the appearance of a desired phenotype. This knowledge is necessary in order to be able to make targeted and rationally selected genetic modifications. However, a variety of cellular properties important for the industry and research, such as resistance to organic solvents and optimal production of certain metabolites, are still poorly characterized and frequently arise from modifications in pathways that involve genes of unknown function or ones that would be impossible to predict by rational engineering, possibly because they act by indirect or compensatory mechanisms. In these cases, where educated guesses about possible interventions are hard or impossible to make, a different strategy can be applied, which is termed “inverse metabolic engineering” [17]. In this type of metabolic engineering, the desired property is first linked to a readily detectable phenotype, e.g. changes in cell growth, color, fluorescence etc. Then, random genetic modifications, such as chromosomal point mutations, gene deletions, gene over-expressions etc., are introduced into the host so that a library of cells with genetic diversity is generated. This library is then screened and the clones that exhibit the desired phenotype are identified. Genetic analysis of the isolated clones subsequently can reveal the factors responsible for the improved properties and lead to enhanced understanding of the (previously unidentified) biochemical processes involved.

Given the availability of an effective genetic screening or selection system for the desired property, the potential success of an inverse metabolic effort is highly dependent on the level and quality of genetic diversity which can be generated. Here, we review combinatorial approaches for generating genetic diversity within a host cell population, and discuss the applications of these methodologies in inverse metabolic engineering. Emphasis is placed on approaches which have been developed in recent years.

## 2. Classical approaches in inverse metabolic engineering

### 2.1. Spontaneous mutagenesis

Spontaneously acquired mutations leading to increased fitness under specific growth conditions is the classical adaptive evolution paradigm. This has been used by mankind for millennia, and by the industry and academia for decades in order to generate useful organisms for a variety of applications. Numerous successes in inverse metabolic engineering mediated through spontaneous mutagenesis have been reported and include increased tolerance to isobutanol and ethanol [18, 19], growth on citrate [20]; production of D-lactate [21] and hard-to-express recombinant proteins in *Escherichia coli* [22]; production of 1,3-propanediol in *Klebsiella pneumoniae* [23]; xylose and galactose utilization in *Saccharomyces cerevisiae* [24, 25]. Analysis of the genetic lesions that lead to improved phenotypes in spontaneously evolved strains used to be a complicated or impossible process for variants carrying multiple and distantly located mutations, but the recent development of -omics technologies coupled with the increased throughput and decreased cost of sequencing technologies has made this a very tractable task [19–21, 26].

### 2.2. Random mutagenesis with chemical mutagens

Libraries of cells containing lesions randomly distributed over the entire chromosome can be readily generated by exposing a population to sub-lethal doses of mutagenic chemicals, such as N-methyl-N′-nitro-N-nitrosoguanidine and ethyl methanesulfonate or other mutagenic agents, such as UV irradiation. The use of such agents has resulted in the development of microbial strains with the ability to produce enhanced amounts of isobutanol [27], full-length IgG antibodies [28] and membrane proteins [29].

### 2.3. Transposon mutagenesis

Genes whose products have a negative impact on a desired property can be readily identified by transposon mutagenesis. This type of mutagenesis results in random insertions of transposable elements throughout the genome with concomitant functional disruption of the gene sequence that received the insertion. Transposon mutagenesis has led to the development of improved strains and the identification of inhibitory roles for genes involved in the production of biomass [30], lycopene [31], and membrane proteins [32] in *E. coli*; riboflavin production in *Bacillus subtilis* [33]; and poly-3-hydroxybutyrate in *Synechocystis* sp. PCC6803 [34], to name a few examples. Very useful tools for studying the effect of gene knockouts is the Keio collection, a publicly available library of all single knockouts of all the non-essential *E. coli* K-12 genes [35] and the yeast deletion collection [36]. The utility of these libraries in inverse metabolic engineering has already been demonstrated [37] and it is expected that such libraries will be increasingly used in the coming years.

### 2.4. Gene overexpression libraries

Genes, gene fragments or fragments of entire operons that favorably affect a desired property can be isolated from vector libraries co-expressing genomic fragments. Genomic libraries have been screened in order to identify genes that enhance alcohol tolerance/production and galactose fermentation in *S. cerevisiae* [38–40]; acetate and butanol tolerance [41, 42], lycopene [43] and membrane protein production [44] in *E. coli*; butyrate tolerance in *Clostridium acetobutylicum* [45], and in other cases. In addition, individual enhancer genes can be identified using the ASKA library, a library of all the *E. coli* open reading frames (ORFs) transcribed from the strong and inducible T5*lac* promoter [46] or the FLEXgene collection, an analogous library encoding yeast ORFs from *S. cerevisiae* [47], both of which are publicly available. Again, such collections have already been used successfully for inverse metabolic engineering applications [48, 49]. To explore the functional genomic content of unculturable organisms, similar screens can also be carried out by constructing and screening metagenomic libraries [50].

Finally, additive positive effects from genes and/or operons distantly located within a genome can be identified by a recently developed tool termed coexisting/coexpressing genomic libraries (CoGeLs) [51]. CoGeLs allow the simultaneous screening of two genomic libraries encoded in different vectors with compatible origins of replication which can coexist in the same host. These vectors can be regular bacterial plasmids that contain small- or medium-size inserts (up to 10 kbases) or fosmids, which are low-copy number vectors where large DNA fragments (∼40 kbases) can be inserted. Using a combination of two plasmid-encoded *E. coli* genomic libraries, Nicolaou et al. demonstrated that CoGeLs can be used to identify combinations of distantly located factors that impart increased acid resistance in *E. coli* [51].

## 3. Recently developed approaches in inverse metabolic engineering

In recent years, a number of new approaches have been developed that attempt to create more “global” changes on cellular pathways and physiology as means of generating complex phenotypes more effectively. The majority of those strategies aim at modifying the transcriptional landscape of an organism, e.g. by generating libraries of randomized transcription factors or by mutating components of the RNA polymerase. Since certain components of the transcriptional machinery in prokaryotic as well as eukaryotic organisms regulate the expression of a wide repertoire of genes, subtle changes in these components can have a pronounced effect on the transcriptome of the cell, thus offering the potential for the emergence of diverse and complex phenotypes [52]. Some examples of these methods are described below and are summarized in Table 1.

**Table 1:** Combinatorial genome engineering approaches which have been applied to inverse metabolic engineering applications.

Method	Targeted cellular component	Target organism	Engineered phenotype	References
Spontaneous chromosomal mutagenesis	Chromosome	*Escherichia coli*	Ethanol and isobutanol tolerance; D-lactate, and hard-to-express protein production	[18, 19, 22]
		*Saccharomyces cerevisiae*	Xylose consumption	[25]
		*Klebsiella pneumoniae*	1,3-propanediol production	[23]

Chromosomal mutagenesis using chemical mutagens or mutator genes	Chromosome	*E. coli*	isobutanol, membrane protein, and full-length IgG production	[27–29]

Transposon mutagenesis	All individual chromosomal genes	*E. coli*	Biomass, lycopene, and recombinant membrane protein production	[30–32]
		*Bacillus subtilis*	Riboflavin production	[33]
		*Synechocystis PCC6803*	Poly-3-hydroxybutyrate production	[34]
		*S. cerevisiae*	Isoprenoid production	[37]

Genomic libraries and related approaches (individual gene overexpression libraries, CoGeLs)	Chromosomal fragments	*E. coli*	Acetate, glutamate, butanol, antibiotic and toxin tolerance; lycopene and membrane protein production	[41–44, 48, 49, 51]
		*S. cerevisiae*	Alcohol tolerance and production; galactose fermentation	[38–40]
		*Clostridium acetobotulicum*	Butyrate tolerance	[45]

Global transcription machinery engineering (gTME)	General sigma factor σ^70^, stationary phase sigma factor σ^S^, RNA polymerase α subunit, cAMP receptor protein (CRP), histone-like nucleoid structuring protein H-NS, H-NS-interacting haemolysin expression modulating protein Hha, *Deinococcus radiodurans* global regulator IrrE	*E. coli*	Ethanol, butanol, isobutanol, pentanol, 3-pentanol, acetate, butyrate, high osmolarity, and SDS tolerance; lycopene, L-tyrosine, and hyaluronic acid production	[61, 66, 67, 69, 71–73, 75, 76, 109]
	Transcription factor Spt15p and TATA-binding protein Taf25p	*S. cerevisiae*	Ethanol tolerance and production; xylose fermentation; corn cob acid hydrolysate tolerance	[67] [70, 74]
	General sigma factor RpoD	*Lactobacillus plantarum*	lactic acid and hydrochloric acid tolerance	[68]

Libraries of artificial zinc fingers	Zinc finger domains fused to transcriptional activators, repressors or without fusion partner	*S. cerevisiae*	Tolerance to heat and osmotic stress; ketoconazole resistance	[57]
		Mouse neuroblastoma cells	Neurogenesis, differentiation of neuroblasts to osteoblasts, proliferation rate	[57]
		*E. coli*	Tolerance to butanol, heat, cold, and osmotic stress	[58–60]

Multiplex automated genome engineering (MAGE)	Multiple rationally selected genomic loci	*E. coli*	Lycopene and indigo production; incorporation of artificial amino acids	[79, 80, 84]

Trackable multiplex recombineering (TRMR)	>95% of all individual *E. coli* genes	*E. coli*	Tolerance to salicin, D-fucose, methylglyoxal, valine, acetate and lignocellulosic hydrolysate	[85, 86]

Ribosome engineering	Ribosomal components or RNA polymerase subunits	*Streptomyces* strains	Actinorhodin, fredericamycin, formycin, actinomycin, piperidamycin production	[88–92, 96]
		*B. subtilis*	Amylase and protease production	[93]
		*Pseudomonas putida*	Resistance to toluene, *m*-xylene, and 4-hydroxybenzoate	[97]

Genome shuffling	Chromosome	*Streptomyces fradiae*	Tylosin production	[100]
		A strain of *Lactobacillus*	Tolerance to lactic acid	[102]
		*Sphingobium chlorophenolicum*	Degradation of pentachlorophenol	[110]
		*E. coli*	Butanol and antibiotic tolerance	[104, 107]
		*S. cerevisiae, Pichia stipitis*	Ethanol production	[105]

### 3.1 Zinc finger-based artificial transcription factors

Zinc fingers are highly specific DNA-binding protein domains which recognize three-base pair sequences and are found in many proteins that regulate transcription in a variety of organisms. One transcription factor can include several of these motifs. The ones that contain more fingers recognize larger stretches of DNA and are, generally, more specific about the genomic loci that they bind. Currently, at least one natural or engineered zinc finger exists for every possible triplet of DNA bases (4x4x4 = 64 possible triplets) [53–55]. Park et al. took advantage of the wide repertoire of DNA-binding specificities and the highly modular way with which these proteins can be assembled [56] to generate libraries of artificial transcriptional activators and repressors that can activate or silence practically any gene within a eukaryotic genome [57], with the ultimate goal of evolving complex phenotypes. First, they selected 40 and 25 zinc fingers with diverse DNA-binding specificities and used them to create random combinatorial combinations of three- and four-finger proteins, respectively. They then fused these randomly assembled finger triads and quadrads to a transcriptional activation domain, a transcriptional repressor domain, or no domain, to generate a library of artificial factors which can potentially regulate the transcription of genes close to their DNA-binding site. Expression of the generated three- and four-finger libraries fused to the Gal4 activation domain, the Ume6 repression domain, or no domain in *S. cerevisiae* resulted in the emergence of novel phenotypes such as growth arrest, and resistance to heat, osmotic stress, and the mycocidal antibiotic ketoconazole [57]. Replacement of the activation and repression domains with p65 and the Krüppel-associated box (KRAB), expression of the transcription factor library in mammalian cells, and screening for the emergence of complex phenotypes resulted in the identification of (i) one p65-containing zinc finger activator, which could induce neurogenesis of the mouse neuroblastoma cell line Neuro2A; (ii) one zinc finger activator that induced the differentiation of murine myoblasts to osteoblasts; and (iii) one inhibitor and one enhancer of cell proliferation [57]. Artificial zinc finger transcription factors can also be used to impart novel phenotypes in lower organisms such as bacteria, either in the absence of a transcriptional regulation domain [58], or in the presence of an activator, such as the cAMP receptor protein (CRP) [59, 60].

### 3.2 Global transcription machinery engineering (gTME)

gTME is another powerful new tool that enables the reprogramming of the cellular transcriptome through random mutagenesis of specific components of the global transcriptional machinery of a microbial organism. Initially, the components which were selected for mutagenesis were the *E. coli* general sigma factor σ^70^ [61], and the *S. cerevisiae* TATA-binding transcription factor Spt15p and the TATA-binding protein associated factor Taf25p [62]. The rationale behind the choice of σ^70^ as a target was that it regulates the expression of about 1,000 genes, which are important for normal exponential growth in bacteria [63], while missense mutations in *rpoD*, the gene encoding σ^70^, have been shown to result in changes in the binding preferences of the RNA polymerase-σ factor complex [64, 65]. Similarly, Spt15p and Taf25p were chosen because they are regarded as two of the main DNA-binding proteins that regulate the promoter specificities of the three RNA polymerases in yeast. These factors were mutated by error-prone PCR to form combinatorial libraries of random mutant alleles, cloned into appropriate vectors, and expressed in *E. coli* and yeast strains that contained the native genomic copy of the corresponding factor. After performing relevant genetic screens and selections, variants of the targeted factors were identified, whose expression resulted in the emergence of industrially important properties. In *E. coli*, several multiple point and truncation *rpoD* mutants were isolated that conferred increased tolerance to ethanol (up to 60 g/L), to sodium dodecyl sulfate (SDS), and to both ethanol and SDS, as well as enhanced production of lycopene (10-50% increase depending on the parental strain) [61]. In yeast, a triple point variant of Spt15p was isolated that exhibited a 13-fold increase in growth at specific glucose concentrations and a volumetric production of ethanol enhanced by ∼70% compared to the parental *S. cerevisiae* strain [62].

After this initial success, a number of reports described the use of gTME to evolve bacterial and yeast strains exhibiting an array of industrially important phenotypes and properties, such as enhanced production of hyaluronic acid [66], L-tyrosine [67], and lactate [68]; increased to tolerance to ethanol, butanol, acetate, butyrate, lignocellulosic hydrolysates, and osmotic stress [69–73]; improved capacity to ferment xylose [74]; and regulated biofilm formation and dispersal [75, 76]. Furthermore, these studies showed that appropriate protein targets for gTME are not limited to σ^70^, Spt15p, and Taf25p, but can also be applied to additional components of the transcriptional machinery of an organism which can simultaneously affect the transcriptional profile of multiple genes, such as the α subunit of the *E. coli* RNA polymerase [67], CRP [69, 72], the histone-like nucleoid structuring protein H-NS [76], and the H-NS-interacting haemolysin expression modulating protein Hha [75]. In certain cases, it may be beneficial to target more than one of these factors simultaneously [77].

It is interesting to mention that improved cellular properties can be engineered not only by targeting endogenous global transcriptional regulators, but also by applying gTME to targets derived form heterologous hosts. For example, Chen et al. isolated variants of the global regulator IrrE from *Deinococcus radiodurans* (a bacterium with very high levels of resistance to radiation) that enhanced stress tolerances when expressed in *E. coli* [73]. However, not all global transcriptional regulators are appropriate gTME targets for a given property. This was demonstrated in the case of the *E. coli* stationary phase sigma factor σ^S^, where effective variants for enhancing hyaluronic acid production in *E. coli* could not be identified, despite the fact that multiple enhancer variants could be identified when σ^70^ was subjected to mutagenesis [66]. Of course, one cannot rule out the possibility that σ^S^ variants useful for hyaluronic acid production in bacteria could exist but, due to the limitations in combinatorial protein library construction, they were not present in the screened *rpoS* library. Finally, but very importantly, transcriptional regulators which have been engineered by gTME are not only useful for conferring improved phenotypes to standard laboratory strains such as *E. coli* K12, but also for other microbes of industrial interest, such as *Lactobacillus plantarum* [68]. Once functional variants of gTME targets have been identified, the changes within the transcriptional landscape responsible for the appearance of the beneficial properties can be identified by DNA microarray analysis.

Based on the data described above, one can now claim that gTME is a relatively well established approach for inverse metabolic engineering applications. Through the already generated results, a number of important conclusions have been drawn, which will provide useful guidance for future gTME efforts. For example, Stephanopoulos and co-workers have tested gTME target libraries generated by low, medium, and high mutagenesis frequencies and have proposed that high-error rate mutagenic PCR pools exhibit increased probability to contain effective variants [68, 71]. This is consistent with the outcome from previous directed protein evolution efforts [78] and with the results of the analysis of the transcriptional regulator variants evolved by gTME, which has shown that the emergence of significantly improved phenotypes usually requires multiple mutations [61, 62, 66, 72, 73]. An interesting observation is that the effective variants are very often N- or C-terminal truncations of the original regulator [61, 66, 67, 71, 77]. Since improved phenotypes upon expression of the truncated regulators occur in the presence of the full-length chromosomal copy of the regulator gene, it is likely that the engineered properties arise from the combined action of the evolved truncation and the full-length, wild-type protein [67]. Alternatively, the evolved truncations could be acting as anti-sigma factors [77]. In cases where a truncated factor is responsible for the emergence of the desired phenotype, it may be more efficient to perform random mutagenesis using the truncated rather the full-length form of the protein [71].

As anticipated, the effects of the evolved transcriptional regulator proteins are phenotype-specific, i.e. a regulator evolved to impart a specific property will, in general, be ineffective in improving another desirable phenotype. However, in cases where different phenotypes are linked to similar cellular pathways, regulators evolved for one phenotype may have similar effects on another. For example, mutants of *rpoA* (the gene encoding the α subunit of the *E. coli* RNA polymerase) that resulted in enhanced resistance to 1-butanol were also found capable of enhancing bacterial resistance to isobutanol, 1-pentanol, and 3-pentanol [67]. For applications where two different unrelated cellular properties need to be simultaneously improved, an effective regulator for both properties can be evolved sequentially (first under conditions which are selective for property A and then the isolated variant can be mutated again and be subjected to selection for property B) or simultaneously (under conditions which are selective for both A and B) [61].

### 3.3. Multiplex automated genome engineering (MAGE)

**Figure 1:**
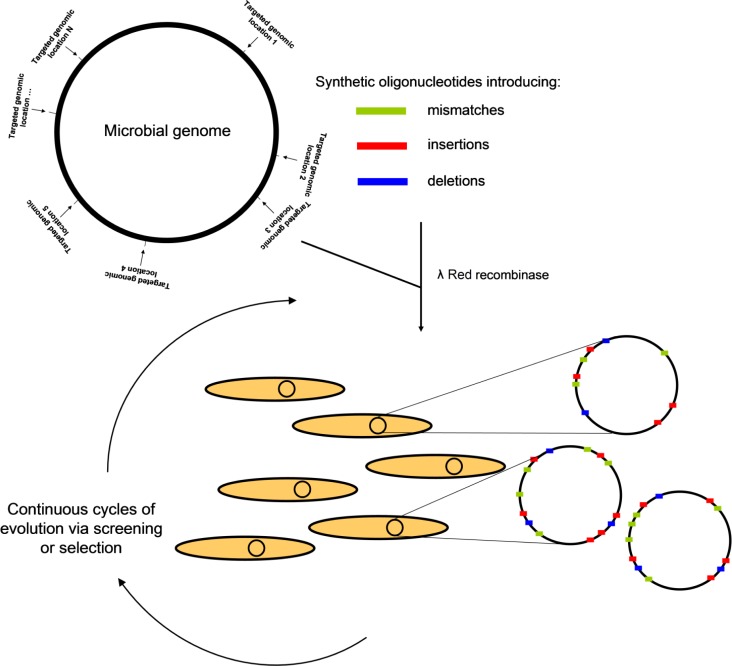
Schematic representation of the MAGE approach. First, specific genes/genomic locations known/suspected to be involved in a particular cellular phenotype are targeted for modification. Then, synthetic oligonucleotides that introduce insertions, deletions, missense mutations, or other types of genetic lesions are synthesized and introduced into the target cell host by electroporation. Subsequently, the action of the λ Red recombinase system assists the introduction of the designed lesions into the target genomic loci. Finally, the beneficial mutations are selected and enriched by performing cycles of genetic screening or selection. The beneficial genomic alterations can be readily identified by DNA sequencing of the targeted genomic locations.

One of the most promising recent genome engineering approaches is MAGE [79]. A key advantage of this technology is that it is capable of generating high levels of genetic variability within multiple genomic loci simultaneously. MAGE utilizes synthetic single-stranded DNA oligonucleotides and the Redα/Redβ recombinase system from bacteriophage lambda in order to introduce point mutations, insertions, and deletions into targeted genomic loci of a microbial organism, such as *E. coli* (Figure 1). Synthetic oligos carrying homology regions with the targeted genomic regions along with the desired modifications are electroporated into modified bacterial strains, which are deficient in the DNA mismatch repair gene *mutS* and which express the components of the λ Red recombinase system. By performing repeated cycles of this process, at least one targeted region can be modified in approximately 30% of the cells of a bacterial population in a period of time as short as two hours [79]. By utilizing prototype mutagenic oligos, Wang et al. estimated that they could generate a population that contained close to 10^10^ variations in sequence compared to the parental cells within a day. The efficiency with which a particular modification is made depends on the degree and length of the homology region between the genomic target and the mutagenic oligo. Continuous MAGE cycles can be carried out until the desired genetic diversity is achieved, a factor which depends on the number of the genomic loci that will be modified and the degree of sequence diversity that will be introduced at each locus. Church and co-workers have already built a prototype device that automates the entire process, thus allowing rapid and easy generation of cell populations with very high levels of genetic diversity [79]. As the prices for custom oligonucleotide synthesis decrease, the applicability of this technology has the potential to become very broad.

**Figure 2:**
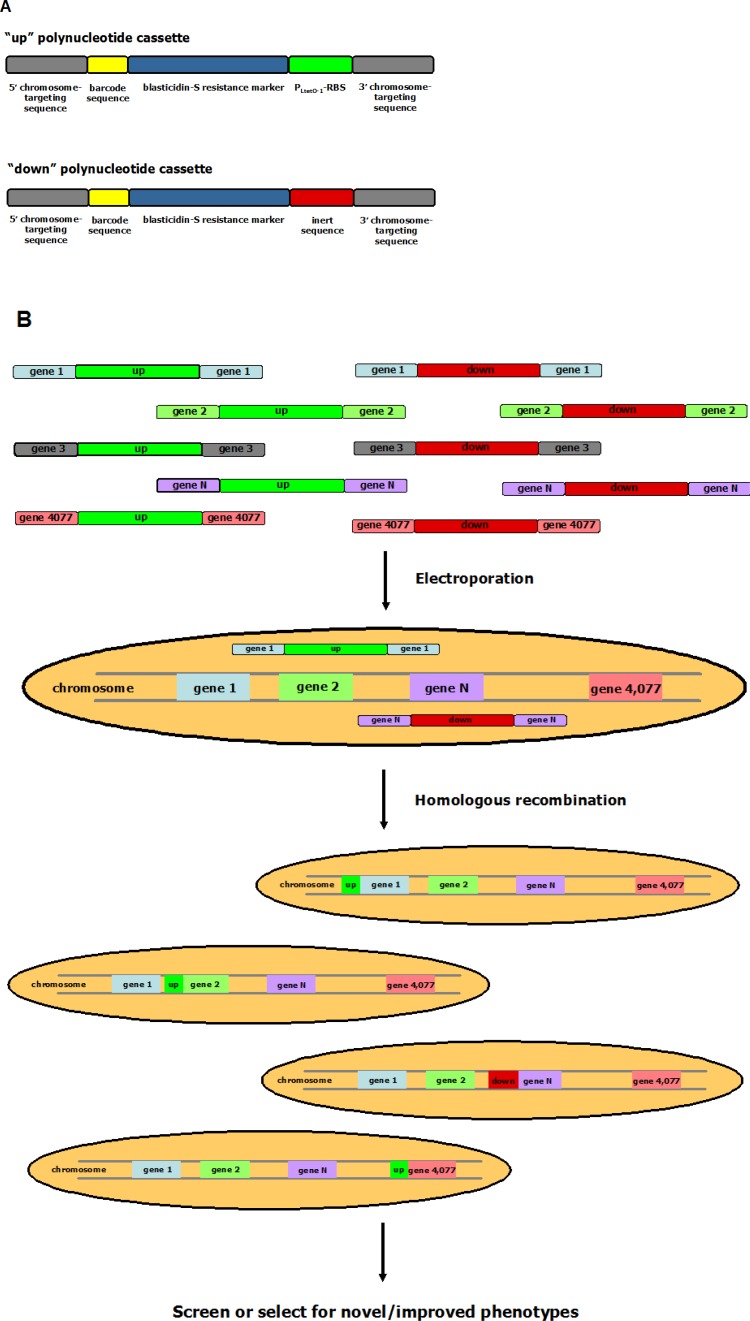
Schematic representation of the TRMR approach. **(A)**. Synthetic DNA oligos in TRMR used for targeted gene up- and down-regulation in a microbial organism. These include gene-specific 5′- and 3′-terminal homology regions (grey), a gene-specific molecular barcode sequence (yellow), a blasticidin-S resistance marker for selecting for cells where successful recombination has occurred (blue), and a sequence which results, in general, in up- (green) or down-regulation (red) of the gene located immediately downstream from this sequence. The sequence responsible for the up-regulation of gene expression contains the strong and inducible P_LtetO-1_ promoter [108] and a ribosome-binding site (RBS), while the sequence responsible for gene down-regulation contains an inert sequence. **(B)**. Gene-specific synthetic oligos the lead to up- and down-regulation were generated for each one of the 4,077 genes of *E. coli* MG1655, yielding a total of 8,154 gene-targeting oligos. These oligos can then be introduced into living microbial cells by electroporation. Successful homologous recombination (selected for by the emergence of a resistance phenotype to the antibiotic blasticidin) can then lead to the creation of a microbial cell library, where each member expresses a different gene at higher or lower levels compared to the parental cells. Genes that have an enhancing or inhibitory role for a particular cellular property can then be identified by subjecting the TRMR-generated library to an appropriate genetic screen or selection, followed by sequencing or microarray analysis.

In order to demonstrate potential applications of MAGE in cellular programming, Wang et al. utilized the approach to enhance lycopene production in *E. coli*. The ribosome-binding sequences (RBSs) of twenty genes previously reported to affect lycopene production were randomized simultaneously, while four other such genes were targeted for inactivation by introduction of internal stop codon mutations. After performing 35 cycles of MAGE and screening for colonies with intense red pigmentation, the authors identified variants carrying RBS sequence modifications in four genes (*dxs*, *dxr*, *idi*, *ispA*) acting at the beginning and the end of the lycopene biosynthesis pathway, while three out of the four targets for inactivation were found to contain stop codons. Some of the isolated mutants were found to produce up to five-fold more lycopene than the parental bacterial strains. This corresponded to a yield of 9 mg/g of dry cell weight, higher than all other previously reported efforts [79]. In addition to enhanced metabolite production, MAGE has additionally been applied to bacterial genomic reprogramming in order to increase the incorporation of artificial amino acids into engineered proteins [80].

More recent improvements of the technology [81–83] have resulted in increased efficiency of the MAGE process, which has allowed for efficient multi-loci insertion of longer sequences (>10 bases) of DNA within a microbial genome (co-selection MAGE or CoS-MAGE) [84]. By using CoS-MAGE, Wang et al. were able to rapidly generate engineered *E. coli*, where the promoters of up to seventeen endogenous genes could be replaced with the strong and orthogonal-to-*E. coli* T7 promoter. Screening for enhanced production of the blue dye indigo in the generated T7 knock-in strains led to isolation of variants which can produce more than two-fold more dye (∼9 mg/g of dry cell weight) than the parental strain [84]. Despite the tremendous potential of MAGE and CoS-MAGE for application in inverse metabolic engineering efforts, one should consider that these approaches can only be applied in cases where genes relevant to a particular cellular property have already been identified previously by other techniques.

### 3.4 Trackable multiplex recombineering (TRMR)

Very recently, Gill and co-workers have developed an elegant methodology for constructing libraries of genetically modified microorganisms based on homologous recombination of pools of synthetic oligos [85]. To demonstrate the technology, the authors constructed two sets of polynucleotide cassettes each of which comprised 5′ and 3′ recognition sequences for homologous recombination with the genomic region immediately upstream of the start codon of each one of the 4,077 protein-coding genes of *E. coli* MG1655, interrupted by a gene-specific tracking sequence (barcode oligoncleotide), a blasticidin-S resistance marker for selection of successful recombination, and either an “up” cassette or a “down” cassette. The “up” cassette consisted of the sequences of the strong inducible promoter P_LtetO-1_ and a ribosome-binding site, whereas the “down” cassette comprised a transcriptionally and translationally inert sequence. The function of the up cassette was to generally up-regulate the expression of its target gene, while the down cassette was intended to down-regulate gene expression. After introduction of this library of polynucleotides into *E. coli* cells by electroporation and selection in antibiotic-containing media for clones where successful homologous recombination took place, the investigators were able to generate libraries of modified bacteria where each member contained a different up-regulated or down-regulated gene. This pool of 2 x 4,077 = 8,154 mutant strains was subsequently subjected to selection for growth under conditions that normally do not permit growth of wild-type *E. coli*. These included media that contain salicin as the sole carbon source, media where the presence of D-fucose inhibits the ability of *E. coli* to utilize arabinose as a carbon source, and lethal concentrations of methylglyoxal (due to extensive oxidative damage), valine (due to feedback inhibition of the biosynthesis of leucine and isoleucine), and lignocellylosic hydrolysate (due to the presence of a cocktail of different growth inhibitors). Warner et al. reported that clones resistant to these conditions could emerge from the described TRMR libraries with frequencies 100-fold higher compared to the same cells lacking the introduced polynucleotide libraries [85]. Subsequently, the isolated clones could be easily characterized by DNA sequencing or microarray analysis using their corresponding molecular barcode sequences to identify the genes responsible for these complex phenotypes. In this manner, already known as well as novel genes involved in the emergence of these cellular properties could be identified.

More recently, the same laboratory demonstrated that the ability of TRMR to assess the effect of up- and down-regulating individual genes on desired cellular properties at a genome-wide scale can be combined with the more in-depth search for the optimal transcription levels of genes relevant to a specific phenotype, which is offered by MAGE [86]. In that work, Sandoval et al. first used the TRMR libraries described above to identify genes which are involved in enhanced bacterial resistance to corn stover hydrolysate, acetate, and low pH, and to rank their relevance in these improved phenotypes. Then, they generated MAGE-like bacterial libraries, where the RBSs preceding the start codons of the genes identified as relevant in the previous step for the property in question were randomized. Based on the calculated efficiency of the process, the authors estimated that they could generate a population that contained a significant percentage of single, double, triple and quadruple mutants (i.e. mutants where the RBSs of one, two, three, and four genes were modified, respectively) after thirteen cycles of recombination. These libraries were then re-subjected to genetic selection for improved phenotypes. In the case of the resistance phenotype to low pH, one clone was identified with improved growth characteristics compared both to the wild-type as well as the single mutant isolated by TRMR [86], thus demonstrating the potential power of combining the two methodologies. In the case of the other two selection conditions which were tested, however, no multi-gene mutant could be isolated that performed better than the single-gene variants identified by TRMR only. This highlighted the importance of epistatic interactions between the different genes associated with a particular phenotype. It remains to be seen whether such combinations of TRMR and MAGE will yield strains with improved characteristics for a variety of cellular properties.

### 3.5 Ribosome engineering

Another very interesting approach for generating complex phenotypes in microbes is “ribosome engineering”. Here, microbial cells are exposed to lethal concentrations of antibiotics that inhibit ribosomal function, such as chloramphenicol, rifampicin, streptomycin, kanamycin, spectinomycin, and others, and clones resistant to different levels of these compounds are selected [87]. Resistance to these antibiotics frequently arises from mutations in components comprising the ribosome, such as ribosomal proteins and RNA, or in other factors that affect mRNA translation, such as elements of the RNA polymerase. These changes can result in aberrant protein synthesis and in enhanced production of specific proteins. In certain cases, some of these overproduced proteins are encoded by genes which are normally in a dormant state. This altered proteome can in turn lead to the emergence of improved or even novel cellular properties. In one of the first demonstrations of the approach, Ochi and co-workers showed that mutations in *rpsL*, the gene encoding the ribosomal protein S12, which confer resistance to streptomycin in *Streptomyces lividans* and *Streptomyces coelicolor* result in increased production of the antibiotic actinorhodin [88, 89]. Similar effects could also be observed with *rpsL* mutations that confer resistance to paromomycin [90]. Spontaneous mutations in *rpsL* can impart resistance to high levels of streptomycin and are rare as they occur with a frequency of 10^−9^-10^−10^ [87]. Low-level resistance to streptomycin can be acquired about 1,000 times more frequently by other types of mutations, such as in the gene *rsmG*, which encodes a 16S RNA methyltransferase [91]. Such mutations can also lead to enhanced antibiotic production and, very interestingly, combination of the identified *rpsL* and *rsmG* mutations results in an additive increase in antibiotic yields [91].

Streptomycin tolerance mutations can have a beneficial effect in the production of other useful products apart from actinorhodin in different bacteria. For example, mutants of *Streptomyces chattanoogensis* could produce up to 26-fold more fredericamycin, while mutants of *Streptomyces antibioticus* and *Streptomyces lavendulae* were able to overproduce formycin and actinomycin [92]. Furthermore, streptomycin-tolerant mutants of *B. subtilis* can accumulate enhanced amounts of enzymes, such as amylases and proteases [93]. Mutations in other translational machinery components, e.g. the gene *rpoB* that encodes a subunit of the RNA polymerase, which confer resistance to antibiotics other than streptomycin, such as rifampicin and gentamicin, can also have enhancing effects in antibiotic production [94]. Very importantly, the presence of mutations of this type in a specific bacterium can lead to the production of antibiotics which are not normally produced by the particular organism [95] or even in the production of completely novel compounds with antibiotic activities [96]. Finally, mutations conferring resistance to streptomycin, rifampicin, and gentamicin can also impart other types of industrially relevant phenotypes, such as enhanced tolerance to organic solvents and chemicals, as exemplified by strains of *Pseudomonas putida* whose growth could be sustained in significantly enhanced concentrations of toluene, *m*-xylene, 4-hydroxybenzoate and others [97]. It should be mentioned that the mutations conferring resistance to the different antibiotics can be combined to provide synergistic effects in the desired phenotypes [98] and introduction of these mutations in industrial microbial strains can be as effective in improving desired phenotypes as in the laboratory organism in which they were originally identified [99].

### 3.6 Genome shuffling

In cases where strains with improved cellular phenotypes have been created by one of the techniques discussed above, whole-genome recombination or “shuffling” can be applied to generate hybrid organisms that combine the beneficial mutations in a synergistic manner to further improve the desired properties [100]. Genome shuffling is typically carried out by a technique termed protoplast fusion [101]. Protoplast fusion involves enzymic removal of the cell wall (e.g. with lysozyme in bacteria), exposure to osmotic stabilizers so as to maintain the protoplast structure intact, and the addition of fusogenic chemical agents, such as polyethylene glycol (PEG), that induce the formation of membrane fusions between the generated protoplasts. Alternatively to the addition of PEG, protoplast fusion can take place more efficiently by electrofusion, i.e. by exposure to low-strength electric fields. It has been shown that repeated rounds of genome shuffling can result in the rapid emergence of complex phenotypes, such as improved production of the antibiotic tylosin from *Streptomyces fradiae* [100], tolerance to low pH of an industrial strain of *Lactobacillus* [102], degradation of pentachlorophenol in *Sphingobium chlorophenolicum* [103], butanol tolerance in *E. coli* [104], production of ethanol in yeast [105] etc. However, genome shuffling in Gram-negative bacteria, such as *E. coli*, is rather inefficient [104, 106] and, thus, the technology needs to be improved before it can become one of widespread use.

Kao and co-workers have recently attempted to address this issue by employing the fertility factor plasmid F to generate a system of continuous “sexual” exchange of genetic material between cells of a normally asexual organism, such as *E. coli* [107]. In this system, *E. coli* cells were engineered to become chromosomal DNA donors by incorporating the F factor into their genome along with a pair of sequences that act as origins of transfer, while deletion of the genes *traS* and *traT*, which encode for the proteins TraS and TraT that mediate surface exclusion, increased significantly the frequency of mating and DNA transfer. Winkler and Kao demonstrated that the emergence of a complex phenotype which can arise from mutations in multiple genomic loci that have a small beneficial impact, such as resistance to chloramphenicol, can be accelerated significantly in this system [107]. An important advantage of this approach is that evolution can be carried out continuously in liquid culture, a feature that makes the process considerably easier than classical genome shuffling.

## 4. Outlook

Numerous studies have demonstrated that inverse metabolic engineering can be a very powerful approach for generating engineered strains with complex cellular properties. The availability of a variety of methods that allow the generation of large libraries of mutant organisms carrying different types of genetic profiles holds the potential to greatly expand the repertoire of phenotypes which can be accessed. Analysis of the generated variants will subsequently provide a better understanding of the cellular processes which are critical for the emergence of the desired properties. The acquired knowledge can then be used as a starting point for guiding targeted pathway modifications through classical metabolic engineering or for additional rounds of inverse metabolic engineering, until improvements reach a high enough level to become attractive for academic and industrial applications.
